# Implementation of a Zebrafish Health Program in a Research Facility: A 4-Year Retrospective Study

**DOI:** 10.1089/zeb.2015.1230

**Published:** 2016-07-01

**Authors:** Ana C. Borges, Nuno Pereira, Maysa Franco, Liliana Vale, Margarida Pereira, Mónica V. Cunha, Ana Amaro, Teresa Albuquerque, Manuel Rebelo

**Affiliations:** ^1^ Instituto Gulbenkian de Ciência, Oeiras, Portugal.; ^2^ ISPA—Instituto Universitário, Lisboa, Portugal.; ^3^ Faculty of Veterinary Medicine, Lusófona University, Lisbon, Portugal.; ^4^ Centre for Ecology, Evolution and Environmental Changes, Faculdade de Ciências, Universidade de Lisboa, Lisbon, Portugal.; ^5^ Biosystems and Integrative Sciences Institute, Faculdade de Ciências, Universidade de Lisboa, Lisbon, Portugal.; ^6^ INIAV, IP- Instituto Nacional de Investigação Agrária e Veterinária, Oeiras, Portugal.

## Abstract

In the past two decades, zebrafish (*Danio rerio*)-based research has contributed to significant scientific advances. Still, husbandry and health programs did not evolve at the same pace, as evidenced by the absence of general guidelines. Health monitoring is essential to animal welfare, to permit animal exchanges across facilities, to contribute to robust experimental results, and for data reproducibility. In this study, we report a health program implemented in a zebrafish research facility to prevent, monitor, and control pathogen, and disease dissemination. This program includes quarantine, routine health screening of sentinels, and nonroutine screenings of retired animals and sick/moribund individuals. An extensive list of clinical signs, lesions, and pathogens was monitored based on: daily observation of fish, necropsy, histology, and bacterial culture. The results indicate that the combined analysis of sentinels with the evaluation of sick/moribund animals enables a comprehensive description not only of pathogen prevalence but also of clinical and histopathologic lesions of resident animals. The establishment of a quarantine program revealed to be effective in the reduction of *Pseudoloma neurophilia* frequency in the main aquaria room. Finally, characterization of the colony health status based on this multiapproach program shows a low prevalence of lesions and pathogens in the facility.

## Introduction

The Instituto Gulbenkian de Ciência (IGC) is a research institute devoted to basic biological and biomedical research and committed to promote organism-centered science. The Institute has facilities for model organisms such as mice, rats, zebrafish, frogs, and fruitflies. The zebrafish (*Danio rerio*) facility was established in 2005 to support the developmental biology research program and was incorporated in the Animal House Core Facility (AHCF) in 2009. The AHCF seeks to integrate the management of several animal facilities, namely by sharing technological development and good practices among different animal models within the IGC. The integration of the zebrafish unit in this core facility aimed to adapt well-established practices followed in the rodent facility, including husbandry routines, staff specialization, services, and a health program. This last topic was practically absent from the zebrafish field and turned out to be one of the most challenging aspects that the AHCF had to deal with due to the paucity of available information and lack of awareness among the zebrafish community.^1,2^ In 5 years, the IGC zebrafish facility grew from 60 to 800 tanks (c.a. 14,000 animals), providing animals and services to over 30 researchers. It currently serves four distinct research areas: Aging and Disease, Social Behavior, Developmental Biology, and Organ Regeneration. The growth and diversity of the IGC zebrafish facility reflects the global trend of zebrafish-based research expansion to virtually all areas of biology.^3^

Being aware of the challenges of the rapidly evolving zebrafish field, the AHCF established a dedicated team to run the facility, composed of a manager, technicians, and a veterinarian with specific expertise in aquatic animal medicine. Zebrafish health and biosafety are priority areas for the management team since it is known that animal welfare impacts research outcomes.^4^ A health program was gradually implemented since 2010, consisting of a set of policies and protocols whose goals were to prevent introduction and dissemination of fish diseases, and to monitor pathogen and disease prevalence.^5,6^ An extensive list of clinical signs, histopathologic lesions, and pathogens was monitored in three sampling groups between 2012 and 2015. The groups included not only prefilter sentinels but also apparently healthy retired animals and diseased animals.

We herein report the results of this retrospective study whose goals were as follows: (1) to maximize disease and pathogen prevalence surveillance; (2) to characterize the colony health status; (3) to assess quarantine efficacy in preventing the spread of infectious agents; and (4) to determine age-related conditions.

## Materials and Methods

### Zebrafish facility overview and housing

It is composed of two physically separated rooms: the main aquaria room (also referred to as main room) and quarantine. The main aquaria room has a capacity for 700 tanks distributed by two multilinking WTU systems (Tecniplast^®^) and four ZebTec™ (Tecniplast^®^) stand alone systems (i.e., total volume of system 1: 1800 L; system 2: 800 L; each stand-alone: 250 L). The recirculation systems have a built-in filtration system composed of a mechanical cartridge of 50 μm, activated carbon filter, and UV lamp (power: 120,000 μW/cm^2^/s). Mechanical cartridges and carbon filters are replaced every 750 h. This room holds experimental animals, breeder stocks, and a nursery area. A zebrafish procedure room is available within the main aquaria area to minimize the need to take animals out of the facility. It is equipped with two microinjection workstations, a fluorescent imaging acquisition station, temperature controlled chambers, a water bath, a scale, a fume hood, bench space, and a computer. The quarantine area is composed of a single room located in a separate building. It is equipped with a Marine Biotech Z-Mod^®^ Aquaria rack system with capacity for 126 tanks. This recirculation system has a mechanical cartridge filter and UV lamp (225,000 μW/cm^2^/s). UV lamps are replaced every 9,000 h. Fish are kept on a 14h light/10h night cycle.

### Water chemistry

In the main room, life support systems are equipped with real-time readers of pH, conductivity, and temperature. These parameters are recorded daily and confirmed weekly with independent pH and conductivity probes (pH meter pH-20 and 0EC/TDS/Temp COM-100 manufacturer: HM digital). Salt (Instant Ocean–Aquarium Systems) is automatically pumped up and dosed at a conductivity of 750 μS/cm (accepted range 650–850 μS/cm). Sodium bicarbonate (Acros Organics) solution is automatically dosed at pH 7.0 (accepted range 6.8–8.5). Water replacement is 10% of total volume per day. Temperature is maintained at 28°C (accepted range: 27°C–29°C). In addition, we perform weekly commercial kit tests for ammonia, nitrites, and nitrates (JBL kit). Accepted ranges: ammonia <0.1 mg/L; nitrites <0.2 mg/L; nitrates <50 mg/L.^7,8^ In the quarantine, pH and conductivity are adjusted manually using the above-mentioned probes. The same water parameters and tolerance ranges are used. All zebrafish life support systems are supplied with reverse osmosis (RO) water.

### Diets

Zebrafish are fed with a combination of live feeds (*Paramecium caudatum* and *Artemia salina*) and processed dry feeds. The detailed feeding regimes, as well as nursery management, are described in Supplementary Data (Supplementary Data are available online at www.liebertpub.com/zeb).

### Health program

**FIG. 1.**
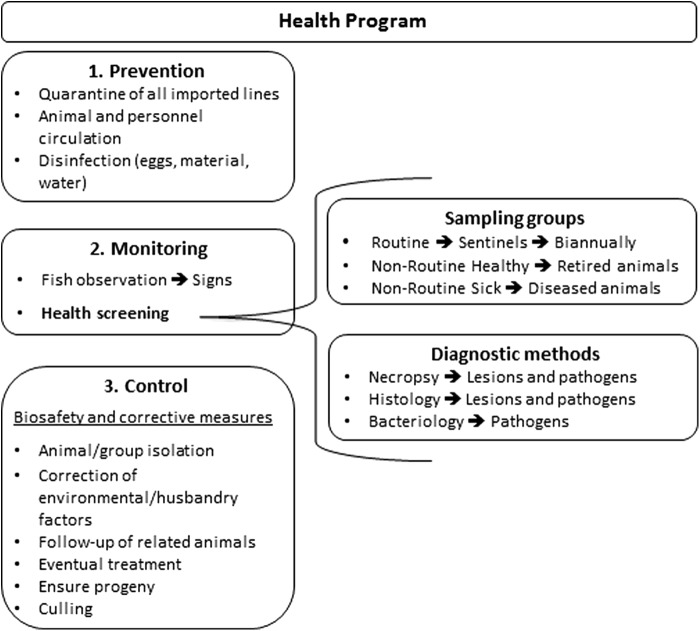
Health Program Overview.

A schematic representation of the health program is shown in Figure 1.

#### Quarantine

The quarantine receives procured animals from other research institutions and stock centers. The AHCF centralizes all importation processes. Only surface disinfected eggs (also referred herein as bleached eggs) enter the housing system of the quarantine (also referred to as quarantine level-1). For this, we rely on the disinfection protocols of sender institutions. As soon as these fish develop to sexual maturity, breedings are set and fertilized eggs are collected, bleached, and transferred to the main room. In addition, adult zebrafish can enter the quarantine and are maintained in quarantine level-2. This is a defined area within the quarantine room equipped with individual life support systems with pump, sediment filter, heater, and biofilter (hobbist-type setup). In this area, biocontainment procedures apply, namely the use of dedicated tanks and nets.

#### Egg surface disinfection

All eggs are surface disinfected before entering any system, both in the quarantine and in the main room (including injected embryos). Briefly, 24–28 h postfertilization (hpf), embryos are immersed in a 36 ppm bleach solution (Sodium Hypochloride 10%–15% - Sigma) in a homemade E3 medium for 5 min with gentle stirring, then rinsed in E3, and submerged in a new 36 ppm bleach solution for 5 min, followed by thorough rinse in the E3 medium.^9^

#### Fish circulation

In the main room, fish are allowed to be transferred between different housing systems, with the exception of a stand-alone rack dedicated to the aging research program (the analysis of this specific group was excluded from this study). Occasionally, some fish are required to be manipulated outside the main aquaria room. In these cases, an “exit-only” policy was defined in which fish are not allowed to come back to the main room, to prevent introduction of potential infectious agents. If these animals are required to be maintained after manipulation, they can be housed in quarantine level-2.

#### Cleaning and disinfection

Tanks, siphons, baffles, and other tank accessories are washed and disinfected in a dedicated washing room by immersion in a 4 ppt bleach solution for 30 min, followed by manual scrubbing, rinsed with RO water in a dishwasher machine at 93°C for 10 min, and dried in a 100°C chamber. Tank cleaning and replacement occurs every 6–8 weeks. Racks are partially disassembled twice per year, pipes and gutters are manually scrubbed and washed with pressurized hot tap water, followed by RO water. Nets are disinfected between each usage by immersion in NetSoak (prepared according to manufacturer's instructions; Jungle Laboratories Corporation) for 1 h, rinsed with RO water, and left hanging until completely dried.^10^ Breeding tanks are washed in a dishwasher machine (program: 3 min at 80°C, 9 min at 48°C, 7 min at 36°C, and RO water rinse 1 min at 75°C). Quarantine material undergoes the same cleaning and disinfection process in a different washing room to avoid cross-contamination. All cleaning and disinfection protocols were validated in-house with the use of an ATP luminescence kit (LuciPac Pen for lumitester PD-20/PD-30 by Kikkoman). In all cases, relative light unit counts decreased more than 99%.^10^

#### Footbaths and personal protective equipment

The entrance and exit of each room are done through a disinfection footbath (The Low Wall Rubber Disinfection Mat from SYNDEL) filled with 10 g/L Virkon Aquatic (Dupont™, Virkon^®^ Aquatic), which is replaced twice per week. The use of gloves is mandatory for all procedures performed inside the zebrafish rooms.

#### Fish observation routine

A daily tank inspection is performed by fish technicians to detect signs of stress and/or disease, as well as to collect moribund and dead animals. A checklist of clinical signs (available in Supplementary Table S1) is used to record the clinical history, and all cases are reviewed by the veterinarian.

#### Routine screening (Routine)

In the main room, between 2012 and 2013, arbitrarily picked animals from each housing system were screened (3–9 fish per rack; ages ranging from 3 to 25 months old). In 2014, sentinel tanks with effluent water circulating from the respective sump to the tank were installed in each of the six life support systems of the main room. Three to four fish per tank (A/B strain) were exposed during 6 months and analyzed at 12 months of age. In the quarantine, routine tests were done on arbitrarily chosen animals from the housing system, since sentinel tanks were not installed. For each quarantine screening, 18–20 animals of various strains were used. Age of animals varied from 3 to 22 months (Table 1). Routine testing was done biannually.

**Table 1.** Sample Characterization

					*Test methods*		
	*Year/group*		*No. of animals*	*Avg age (mo)*	*Microbiology*	*Necropsy*	*Histology*
Main room	Year	2012	97	12.2 ± 6.8	20	33	68
		2013	106	10.1 ± 6.8	21	34	71
		2014	184	13.3 ± 8.1	21	24	157
		2015	120	14.2 ± 7.8	15	15	112
		Total	507	12.7 ± 7.7	77	106	408
	Group	Routine	189	10.6 ± 5.2	65	80	112
		NR-healthy	114	16.2 ± 9.7	4	7	113
		NR-sick	204	12.8 ± 7.7	8	19	183
		Total	507	12.7 ± 7.7	77	106	408
Quarantine room	Year	2012	29	7.0 ± 3.0	11	12	16
		2013	55	7.2 ± 4.5	15	19	36
		2014	51	10.4 ± 5.0	11	11	38
		2015	51	9.7 ± 8.4	9	8	41
		Total	186	9.3 ± 6.3	46	50	131
	Group	Routine	137	9.2 ± 5.9	43	46	88
		NR-Healthy	23	11.7 ± 6.1	3	3	20
		NR-Sick	26	7.9 ± 7.8	0	1	23
		Total	186	9.3 ± 6.3	46	50	131
TOTAL Main room + Quarantine			693	11.8 ± 7.5	123	156	539

Numbers of animals do not always match numbers of tests because the same specimen may be used for necropsy and microbiology.

NR, nonroutine; No, number; Avg, average; mo, months.

#### Nonroutine healthy screening

This group was composed of apparently healthy retired animals (Table 1). This group also included follow-up cases of previously positive diagnosis for disease or pathogens (could be progenitors, progeny, or cohabitants of positive cases). The analysis of this group was initiated in 2014 and did not follow a defined periodicity, as it was done whenever these animals were available.

#### Nonroutine sick screening

This group was composed of animals that were noted to exhibit signs of disease during routine fish observations. Initiated in 2012, this sampling strategy was intensified in 2014, so most of the cases presented in this study are later than May 2014. These tests did not follow a defined periodicity. Dead animals were rarely tested due to the rapid postmortem autolysis.

#### Diagnostic tests

The available test panel was the same for all groups and consisted of necropsy, histology, and bacteriology. However, necropsy and bacteriology culture were performed more frequently on the routine group. From each housing system of the main room, one animal was necropsied and sampled for bacteriology culture; and two to three animals were processed for histological analysis. Water samples were collected on routine screening. All elements described in the checklist (provided in Supplementary Table S1) were considered in the examination.

#### Necropsy

Recently euthanized animals were subject to external and internal gross examination under the stereoscopic microscope, followed by optical microscopic examination that included wet mounts of cutaneous mucous, gill and fin biopsy, and coelomic organs. Before accessing the coelomic cavity, skin was disinfected with 70% ethanol for 5 min and the excess ethanol was absorbed with paper. A small flap of the left coelomic cavity wall was performed with disinfected surgical material avoiding contact with the area to be sampled. A swab was done for bacteriology testing (see Fish Bacteriology section). After this, the coelomic cavity manipulation was done without any further aseptic concern.

#### Histopathology

Whole fish were fixed in 10% buffered formalin for at least 48 h and paraffin embedded through standard methods. Routine stainings were Hematoxylin and Eosin, Ziehl-Neelsen-Fite (ZN), and Luna.^11^ For each animal, sagittal histological sections were obtained, allowing observation of brain, spinal cord, and gills. One slide/staining/animal was routinely observed for diagnosis. Gram staining was performed if bacterial etiology was suspected based on clinical history, gross lesions, and histological findings. If cutaneous ulcers were present, transverse sections of the whole animal were requested. Alternatively, ulcerated skin was gently dissected and processed as an independent paraffin block. When infection by *Pseudoloma neurophilia* and/or *Mycobacterium* spp. was suspected, additional Luna or ZN stainings were requested, respectively. When ZN staining was positive for acid-fast bacteria (AFB), suspected mycobacteria infection was confirmed by PCR (see Diagnosis of *Mycobacterium spp*. infection cases). All histopathology slides were analyzed by the facility veterinarian assisted by a veterinary pathologist whenever necessary.

#### Fish bacteriology

Coelomic cavity swab was done with a sterile inoculation loop, by contacting only the surface of coelomic organs, mainly the liver and the anterior intestine. To prevent contaminations, culture was done only if inoculation loop and surgical instruments did not contact other tissues other than the sampled ones and there was no spread of externally applied ethanol into the coelomic cavity. If lesions such as cutaneous ulcers and gross lesions of swim bladder or other organs were present, swabs and cultures were performed. Samples were inoculated onto blood agar plates and analyzed at an external laboratory (Instituto Nacional de Investigação Veterinária - INIAV). Growing bacteria were inoculated onto the following: MacConkey agar with crystal violet (for Gram - bacilli), trypticase soy agar (for Gram–and + cocci and bacilli), and TCBS (Thiosulfate Citrate Bile Salts Sucrose) agar (selective for *Vibrio* spp.) at 28°C and 37°C. If no bacterial growth was detected after 48 h of culture, the incubation period was extended up to 72 h to maximize potential detection of *Edwardsiella ictaluri*, a slow-growth bacteria that is a relevant zebrafish pathogen.^12^ All the microorganisms grown on MacConkey were subcultured into TSI (Triple Sugar Iron) and tested for oxidase screening. Based on macro and microscopic morphology and Gram-staining characteristics, identification was done through biochemical characterization using the Analytical Profile Index (API) and API^®^ test strips (bioMérieux), following manufacturer's instructions and established algorithms. Quality control strains were used to interpret and validate each test batch. Results reading and interpretation were done with the aid of the ATB™ Expression reading system (version 2.0; bioMérieux).

#### Water bacteriology

Water samples were collected, 50 mL from each system (tanks and sump), and analyzed at the same laboratory referred to in the previous section. Water samples were centrifuged and the sediment was inoculated following the same protocol described above. Antibiograms were done using the automated ATB (bioMérieux) susceptibility testing system or the agar disk diffusion (Kirby–Bauer) method for each and every bacterial isolate (either from fish or from water).

#### Diagnosis of *Mycobacterium spp*. infection cases

A case was considered suspect when positive for ZN staining or when granulomas were observed at the necropsy or histopathology analysis. Total DNA was extracted from paraffin-embedded tissues and tested by PCR techniques.^13–16^ Detailed protocols are available in Supplementary Data.

#### Control measures

After confirming the presence of disease or pathogen, several measures were implemented, including the following: isolation of the affected animal or the affected group; regular examination of health status; eventual treatment; correction of husbandry-related problems; health assessment of the cohabitants, progenitors, or progeny (follow-up procedure); attempt to breed the affected animal to ensure progeny; and culling of the affected fish. Swimming abnormalities and anorexia were considered signs of suffering and humane endpoints.^17^ In these cases, euthanasia was done by immersion in 250 mg/L buffered MS-222 until cessation of opercular movement (5–10 min).^18–20^

#### Medical records

A paper form was filled in by the veterinarian assisted by the zebrafish facility technicians. The form included animal ID, clinical history, necropsy, bacteriology, histology results, and recommended control measures. Health reports were produced biannually and made available to other facilities whenever requested.

#### Data analysis

Medical records were compiled and analyzed in a Microsoft Excel spreadsheet. Clinical findings were classified as signs, lesions, and/or pathogens (full list in Supplementary Table S1). A case was defined as positive diagnosis for clinical signs, histopathology lesions, and/or pathogens based on clinical history, necropsy, and/or histopathology analysis. The prevalence for each clinical finding was calculated as the frequency of positive cases per total number of tested animals analyzed over the 4-year period. This report describes findings observed between January 2012 and September 2015.

#### Statistical analysis

Computation of chi-square and Fisher's exact test was done using VassarStats: Website for Statistical Computation (www.vassarstats.net). Results were considered significant when *p* < 0.05.

### Ethics and legislation

All animal standard operating procedures were ethically reviewed and approved by the Ethics Committee of IGC and the official entity that regulates the use of laboratory animals in Portugal (DGAV–Direção Geral de Alimentação e Veterinária). All experiments conducted on animals followed the National (Decreto-Lei n 113/2013) and European (Directive 2010/63/EU) legislations, concerning housing, husbandry, and animal welfare.

### Training and accreditation

Researchers and technicians of the AHCF are certified by DGAV. For this, since 2009, the AHCF organizes courses on Laboratory Animal Sciences based on the Federation of European Laboratory Animal Science Associations (FELASA) guidelines. The zebrafish training includes husbandry and breeding; anatomy and necropsy; anesthesia and euthanasia; administration routes; microinjection and embryo staging; health control; and a fish facility tour.

## Results

This report describes clinical and management findings recorded between January 2012 and September 2015. We analyzed 693 animals, 507 from the main aquaria room and 186 from the quarantine (Table 1). An increasing number of animals were tested in the main room: 97 in 2012 and 120 in 2015, peaking in 2014 with 184. This sampling accompanied the facility expansion with an increasing number of independent life-supporting systems. The peak of tested animals in 2014 corresponded to an intensification of sampling of retired animals—NR-Healthy group—which became available during this period. In the quarantine, after 2012, the number of examined animals was roughly stable during subsequent years, as the capacity and occupation of this room remained constant. Animals were on an average older in the main room than in the quarantine (12.7 months versus 9.3 months, respectively). This could be justified by the shorter housing period in the quarantine. Moreover, the average age of the NR-Healthy group was the highest (16.2 months) as it corresponds to retired animals, which were older than the fish from the other groups.

The results of the biannual sentinel Routine health screening of the main room are summarized in Table 2. This group displayed overall low frequencies of disease signs and lesions. The most frequently detected clinical signs were abnormal swimming (1.0%) and emaciation (1.0%), and the most represented lesions were ovarian inflammation (3.6%) and splenomegaly (1.3%, enlarged spleen). Pathogens observed by histology revealed that the most frequent agents were *P. neurophilia* (3.6%) and AFB (1.8%), most likely *Mycobacterium* species. The association of some of these clinical signs with specific pathogens was previously reported in the literature: emaciation is a typical clinical sign of *P. neurophilia* infection^21–24^ and splenomegaly is often found in AFB infection cases.^21^ We could not statistically validate these associations in this sample due to the reduced number of positive cases. Noteworthy, the routine testing did not reveal a significant presence of diseases. To make a more detailed characterization of the colony health status, two additional sampling groups were analyzed.^5^

**Table 2.** Health Screening Results of the Routine Group

		*Results*	
*Clinical finding*		*#pos/#test*	*freq (%)*
Signs	Abnormal swimming	2/192	1.0
	Emaciation	2/192	1.0
	Dorsal scale protrusion	1/192	0.5
Lesions	Ovarian inflammation	4/112	3.6
	Splenomegaly	1/80	1.3
	AFB-negative granuloma	1/112	0.9
	Gill hyperplasia	1/112	0.9
	Muscle fiber atrophy	1/112	0.9
	Neoplasia	1/112	0.9
	Aerocystitis	1/192	0.5
	Egg binding	1/192	0.5
	Gill air emboly	1/192	0.5
	Gill telangiectasia/aneurism	1/192	0.5
	Intestinal dilatation	1/192	0.5
	Cutaneous ulcer	1/192	0.5
Pathogens	*Pseudoloma neurophilia*	4/112	3.6
	Acid-fast bacteria	2/112	1.8
	Flavobacter	1/192	0.5
	Nematode eggs	1/192	0.5

Necropsy and histology analysis from the main aquaria room.

AFB, acid-fast bacteria; pos, positive; test, tested; freq, frequency.

The profile of signs and lesions found in NR groups contrasted with the Routine group (Tables 2–4). In the NR-Healthy group, clinical signs and lesions appeared in relatively low frequencies with the exception of macroscopic granulomas (14.3%), which corresponded to only 1 out of 7 analyzed animals by necropsy. However, *P. neurophilia* (6.2%) and AFB (5.3%) were observed at higher frequencies than in the other groups. As expected in the NR-Sick group, we observed a larger set of disease signs and histopathological lesions. The most represented were abnormal swimming (17.3%), negative buoyancy (9.4%), distended coelomic cavity (8.4%), and dorsal scale protrusion (7.9%). Many others were found and are listed in Table 4. In addition, inflammatory and infectious-related lesions were present at higher levels than in the other groups: aerocystitis (24.8%; swim bladder inflammation/infection), cutaneous ulcers (23.8%), coelomitis (17.8%; coelomic cavity inflammation/infection), and branchitis (16.4%, gill inflammation/infection). Neoplasia frequency was also higher in the NR-Sick group (9.8%) when compared to NR-Healthy (1.8%) and Routine (0.9%) groups. Neoplasia in zebrafish is well described in the literature and the affected organs/tissues found in this study have already been reported previously in the head, intestine, pancreas, kidney, testicle, ovary, and lymphohematopoietic tissue (lymphoma).^25^

**Table 3.** Health Screening Results of the Nonroutine Healthy Groups

		*Results*	
*Clinical finding*		*#pos/#test*	*freq (%)*
Signs	Spinal curvature	1/120	0.8
	Emaciation	1/120	0.8
Lesions	Macroscopic granuloma	1/7	14.3
	Aerocystitis	3/120	2.5
	AFB-negative granuloma	2/113	1.8
	Neoplasia	2/113	1.8
	Coelomitis	2/120	1.7
	Ovarian Inflammation	1/113	0.9
	Intestinal dilatation	1/120	0.8
Pathogens	*Pseudoloma neurophilia*	7/113	6.2
	Acid-fast bacteria	6/113	5.3

Necropsy and histology analysis from the main aquaria room.

**Table 4.** Health Screening Results of the Nonroutine Sick Group

		*Results*	
*Clinical finding*		*#pos/#test*	*freq (%)*
Signs	Abnormal swimming	35/202	17.3
	Negative buoyancy	19/202	9.4
	Distended coelomic cavity	17/202	8.4
	Dorsal scale protrusion	16/202	7.9
	Skin congestion	12/202	5.9
	Skin hemorrhage	12/202	5.9
	Group mortality	11/202	5.4
	Subcutaneous emphysema	1/19	5.3
	Spinal curvature	9/202	4.5
	Emaciation	6/202	3.0
	Clamped fins	3/202	1.5
	Cutaneous mass	3/202	1.5
	Distended coelomic cavity + scale protrusion	3/202	1.5
	Dyspnea	3/202	1.5
	Positive buoyancy	3/202	1.5
	Anal prolapsed	1/202	0.5
Lesions	Aerocystitis	50/202	24.8
	Cutaneous ulcer	48/202	23.8
	Coelomitis	36/202	17.8
	Branchitis	30/183	16.4
	Opaque swim bladder	2/19	10.5
	Neoplasia	18/183	9.8
	Ovarian inflammation	6/183	3.3
	Egg binding	6/202	3.0
	Intestinal dilatation	5/202	2.5
	AFB-negative granuloma	4/183	2.2
	Gill hyperplasia	2/183	1.1
	Muscle fiber atrophy	2/183	1.1
	Thyroid hypertrophy	2/202	1.0
	Cardiac dilatation	1/202	0.5
	Exophthalmitis	1/202	0.5
	Gill air emboly	1/202	0.5
	Gill telangiectasia/aneurism	1/202	0.5
	Overinflated swim bladder	1/202	0.5
	Supersaturation	1/202	0.5
	Testicular hypertrophy	1/202	0.5
Pathogens	Acid-fast bacteria	7/183	3.8
	Macroscopic hyphae	4/202	2.0
	Fungal hyphae	1/202	0.5
	Gram-negative bacteria	1/183	0.5
	*Pseudoloma neurophilia*	1/183	0.5

Necropsy and histology analysis from the main aquaria room.

Overall, the analysis of the main room revealed that apart from *P. neurophilia* and mycobacteria, all other tested pathogens (full list in Supplementary Table S1) were virtually absent or detected at very low frequencies (i.e., *Flavobacter-like* bacteria, nematode eggs, and Gram-negative bacteria; Tables 2–4).

**FIG. 2.**
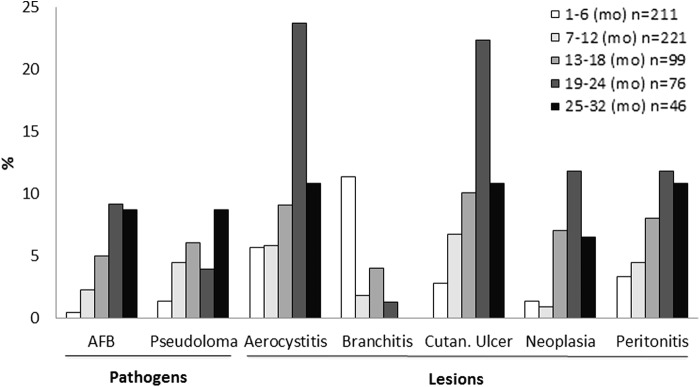
Pathogen and lesion prevalence by age. Data represent the sum of the main aquaria room + quarantine. *n* is the number of sampled animals in each age interval. AFB, acid-fast bacteria; Cutan, cutaneous; mo, months of age.

The rationale for including the NR-Healthy group in this study, which consists of aged animals, was to assess age-related conditions. For this, we calculated the frequency of the most prevalent clinical findings per age interval. These included *P. neurophilia* and AFB infections, aerocystitis, cutaneous ulcers, coelomitis, neoplasia, and branchitis. Figure 2 shows that, with the exception of branchitis, all other depicted lesions increased their prevalence with age, with peaks in the interval of 19–24 months of age.

**FIG. 3.**
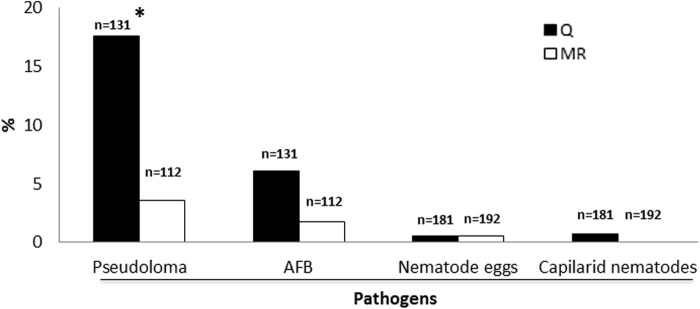
Pathogen prevalence in the quarantine versus main room. *y* axis depicts the percentage of positive cases for pathogens. Q, quarantine; MR, main room. *p < 0.05.

A quarantine program was implemented with the objective of preventing infectious agents from disseminating to the main aquaria room.^5,26^ To assess the efficacy of this strategy, we compared pathogen detection frequencies between the main room and the quarantine (Fig. 3). We observed 17.6% of *P. neurophilia* prevalence in the quarantine versus 3.6% in the main room (*p* < 0.05). Emaciation, a clinical sign associated with microsporidiosis^23,24,27^ and with micobacteriosis,^28,29^ also displayed a reduction: 5.5% in quarantine (Supplementary Table S2) to 1.0% in the main room (Table 2; *p* < 0.05). AFB cases also decreased from 6.1% to 1.8%, although the difference was not significant. All other pathogens analyzed were either absent or present at low frequencies in the quarantine, as depicted in Figure 3 and Supplementary Table S2.

Bacteriological monitoring by characterization of bacterial communities and antibiotic sensitivity (data not shown) is important to assess bacterial disease risk, infection control, and zoonotic hazard.^30,31^ In this study, bacteria were isolated from (1) water tanks and sumps (*n* = 44); (2) coelomic cavities of sentinel fish (*n* = 124); (3) cutaneous ulcers (*n* = 8); and (4) inflamed swim bladder (*n* = 1). Results summarized in Table 5 revealed a dynamic behavior of detected bacterial communities, since the isolated species did not remain constant over the years. We also noticed that the predominant species present in the water were not always associated with those found in fish. In 5 species isolated from cutaneous ulcers, only 2 were also found in the water of the housing system, in the same year: *Aeromonas sobria* and *Shewanella putrefaciens.* To our knowledge, most bacteria isolated in this study have not been described in the literature as zebrafish pathogens, and most of the information about their infectious risk derives from other fish species. Most species described in this study are predominantly opportunistic pathogens that can cause mild to severe infections in immune-depressed animals, depending on chronic stress-inducing factors such as environmental disturbances. We have compiled data from the literature to systematize information about pathogenicity in fish and zoonotic potential of the isolated species^30^ (Table 5).

**Table 5.** Origin of Isolated Bacteria Species

			*2012*			*2013*			*2014*				*2015*		
*Bacteria*	*Pathogenicity*	*Zoonotic risk*	*Water*	*Coelomic cavity*	*Cutaneous ulcer*	*Water*	*Coelomic cavity*	*Cutaneous ulcer*	*Water*	*Coelomic cavity*	*Cutaneous ulcer*	*Swim bladder*	*Water*	*Coelomic cavity*	*Cutaneous ulcer*
*Aeromonas salmonicida* masoucida	F^35,36^	NR^37^					+								
*Aeromonas hydrophila*	Z^38^	Yes^39^		+		+	+			+					
*Aeromonas sobria*	Z^40^	Yes^40^	+	+			+		+		+		+		
*Alcaligenes faecalis*	NR	Yes^*41^	+						+						
*Burkholderia cepacia*	F^42^	Yes^43^												+	
*Escherichia coli*	Z^44^	Yes^$45^	+												
*Plesiomonas shigelloides*	F^31^	Yes^46^					+								
*Pseudomonas fluorescens*	F^31^ Z^47^	Yes^*48^				+					+		+		
*Shewanella putrefaciens*	F^*31^	Yes^*49^		+	+	+	+	+					+		
*Vibrio alginolyticus*	F^22^	Yes^50^		+				+							
*Vibrio metschnikovii*	F^*51^	Yes^52^												+	+
*Vibrio parahaemolyticus*	F^*22^ Z^53^	Yes^22^		+						+		+			
*Vibrio vulnificus*	F^22^ Z^54^	Yes^50^	+							+					
*Weeksella virosa*	NR	Yes^55^											+		

Water, coelomic cavity, cutaneous ulcers, and swim bladder. Results are expressed as present (+) or absent (blank). Pathogenicity column indicates whether bacterial species is pathogenic to Fish (F) or specifically to Zebrafish (Z).

^*^ Rare.

^$^ If pathogenic strain.

NR, not reported.

Among all the bacterial species detected in this study, AFB, most likely *Mycobacterium* spp. are the most predominant in zebrafish facilities.^21,32^ Our primary diagnostic method for these agents is histological staining, which has some technical limitations due to low sensitivity. Therefore, by using PCR-based methods, we attempted to confirm ZN staining results and perform identification at the species level. For this, 26 specimens were analyzed, including 3 with cutaneous ulcers and 3 with AFB-negative histological granulomas. *Mycobacterium peregrinum* and *Mycobacterium marinum* nucleic acids were detected in the coelomic cavities of 2 zebrafish specimens. The *M. marinum* positive test corresponded to a fish from the quarantine. As a follow-up, its progeny was tested and diagnosed AFB negative by histopathology. The *M. peregrinum* case was found in a diseased animal of the main room and displayed abnormal swimming pattern, a cutaneous ulcer, and aerocystitis. Subsequent screenings of sentinels revealed that the infection did not persist. Attempts to discriminate mycobacterial species in 24 additional *Danio rerio* samples, from which genomic DNA had been extracted from the whole paraffin-embedded specimen, were never successful. Further analysis of these samples by real-time PCR allowed the detection of mycobacterial 16S rDNA sequences in 16 out of 24 zebrafish specimens, including the three ulcer cases and one AFB-negative granuloma sample. In these 16 samples, it was not possible to discriminate within the *Mycobacterium* genus, since the genomic region analyzed is highly conserved.

## Discussion

Recommendations for zebrafish health management in research facilities have been proposed,^5^ however, the extent to which these standards have been adopted by individual facilities and resulting outcomes on disease and pathogen burden are largely unknown. In the absence of harmonized guidelines, the evaluation of a single health program is somehow subjective. One would need comparable data from other facilities to validate each program.

We have implemented a zebrafish health program in 2010 and assessed the results over a 4-year period (2012–2015). We analyzed three distinct sampling groups: sentinels/Routine, retired/NR-Healthy, and diseased/NR-Sick. This study allowed us to identify distinct profiles, which may have complementary roles in the health status characterization of a colony. Both the Routine group (composed of prefilter sentinels and arbitrarily chosen animals) and NR-Healthy group (composed of retired animals) showed to be efficient in detecting pathogens, but displayed low disease detection efficacy. By contrast, the NR-Sick group showed to be useful in the characterization of diseases present in the colony, which was relevant to establish management strategies for disease control. Based on previous recommendations further supported by our data, we favor a health program that relies on the combined testing of sentinels and diseased animals to enable monitoring of both pathogen prevalence and disease burden. This strategy also seems to have the advantage of combining routine tests with sporadic analysis of disease cases, bridging the temporal gaps between biannual screenings.

An important aspect of facility management is the estimation of pathogen prevalence in a colony. For this, random sampling and adequate sample size are required, but are often limiting factors in zebrafish facilities.^5,33^ To overcome this, sentinel programs have been proposed with the aim to detect pathogens.^5,6,26^ In this study, the approach taken for pathogen detection evolved progressively. The first phase of routine screening of the main colony was based on arbitrarily picked animals, while the second phase was based on prefilter sentinels. The resulting dataset was merged in a 4-year bulk analysis, therefore, the extrapolation of results to the overall colony must be done carefully. It can be noted that the breakdown into periodic screening results could have been more accurate, yet the combination of sampling groups with the extensive list of clinical findings would have generated an even more complex analysis. Despite these confounding factors, we think that the approach described in this report provides an in-depth description of the IGC zebrafish colony health status.

Of particular concern is the detection of *P. neurophilia* (4/112) and *Mycobacterium* spp. (2/112) in sentinels of the main room, since they are the most widespread zebrafish pathogens in research facilities.^24,32^ The four positive animals for *P. neurophilia* corresponded to only two sentinel cases diagnosed in 2014 and 2015 in two different stand-alone systems. Additional cases diagnosed within the NR groups were isolated and followed up to minimize dissemination. Most of them were successfully handled, as indicated by the absence of positive diagnosis in subsequent health screens. Regarding mycobacteria detection in sentinels, the only two positive cases date to 2014 and have not persisted in the following year. Overall, our interpretation is that these two major pathogens are not widely disseminated in the colony, and control measures have been effective.

The most frequent lesions described in this study, aerocystitis, coelomitis, and cutaneous ulcers, often displayed a clinical presentation with the presence of signs such as abnormal swimming, negative buoyancy, dorsal scale protrusion, skin congestion and hemorrhage, and distended coelomic cavity.^21,22^ As recommended, fish displaying these signs in our facility are isolated, and control measures are applied.^5,21^ Noteworthy, most diagnoses of these lesions and pathogens were found in aged animals (older than 18 months), indicating that an effective age control policy is one of the most important areas of improvement of the IGC facility.

Assessing the efficacy of the quarantine program was another goal of this study. By comparing the pathogen frequency in the quarantine versus the main room, we observed a decrease of both *P. neurophilia* and AFB. Furthermore, we consider that the prevalence of other pathogens is virtually absent from the quarantine, which we attribute to the effective egg-only policy (prequarantine measure), the first barrier in place. However, quarantine results should be interpreted bearing in mind that the Routine group of the quarantine was not housed in prefilter tanks, but rather in conventional ones. This method might be less efficient, eventually leading to an underestimation of infectious agents. Improvement areas in the quarantine include the installation of sentinel tanks, reduction of the animal housing time, and the establishment of a restricted access policy. These measures are already being implemented and we look forward to analyzing results after these changes.

Bacteriological analysis of this study relied on analytical protocols that allowed a qualitative microbiological assessment of potentially pathogenic bacterial species. We have isolated several species from water and animal samples, as an effort to characterize the bacterial communities present in the facility. Some species are known to be opportunistic pathogens and some to be related to zoonotic risk. It was possible to establish some associations between bacteria present in the water and the lesions (*A. sobria* and *S. putrefaciens* in cutaneous ulcers). However, due to the low number of analyzed lesions, it was not possible to establish more robust associations. On the other hand, it could be argued that these bacteria were contaminants from the water. To reduce this possibility, the swabs were done in the ulcer periphery. Regarding zoonotic risk assessment, we recognize the existence of some zoonotic agents; therefore, appropriate measures are in place such as the use of personal protective equipment. Up until now, we have no zoonotic episodes to report. To improve this bacterial profiling, additional sampling of tank biofilm could be tested (either by culture or PCR) since it is known to harbor mycobacteria.^29^ Another interesting direction to explore would be to test water quality indicators for efficacy of water disinfection and water source contamination.^34^

This health program has a strong emphasis on nonmolecular diagnostic techniques that include bacteriology culture, necropsy, and histology, which allow the detection of a large array of zebrafish pathogens. Nevertheless, conscious of the potential low pathogen detection sensitivity of the employed methods, we tried to compensate this fact by increasing the number of sampling groups and incorporating molecular diagnostic techniques for *Mycobacteria* species identification. However, this later analysis was only partially possible due to technical limitations posed by paraffin-embedded samples. Also, the fact that not all AFB-positive cases related to positive PCR amplification of fragments from *Mycobacterium* spp. genome can be due to poor sample quality, PCR inhibition and lower performance with paraffin-embedded tissues or to the remote hypothesis of AFB being attributed to *Nocardia* spp.^22^ Interestingly, the use of PCR to assess *Mycobacterium* spp. in AFB-negative granulomas yielded one positive result (1 out of 3), indicating that even though the histological specimen stained negative for ZN, *Mycobacterium* spp. was present.^32^ This highlights the fact that the observation of granulomas, independent of ZN staining result should be further investigated. As a future direction, we consider that a combination of molecular methods with the currently used analytical tests are worth being further pursued to improve the pathogen detection efficacy of this program.

## Conclusion

The implemented health program in the IGC zebrafish facility in 2010 has been subject to adjustments over the years. Still, there is place for improvements to better control the health status of the colony. In the absence of guidelines for zebrafish health programs, it is highly valuable for facilities to share this type of information to allow mutual assessment. Hopefully, this flow of information could lead to improved practices and standardization of health programs. This step would certainly be beneficial toward the ultimate goal of this exercise, which is to contribute to better zebrafish welfare, robust zebrafish experimental-driven data, and reproducibility.

## Supplementary Material

Supplemental data
