# Supplementary material for: Implementation of a Zebrafish Health Program in a Research Facility: A 4-Year Retrospective Study
Source: Zebrafish. 2016 Jul 1;13(Suppl 1):S-115–26. doi: 10.1089/zeb.2015.1230 (PMC4931741; doi:10.1089/zeb.2015.1230)
Supplement: Supplemental data [file Supp_Data.pdf]

Supplementary Data

Diets and Feeding Regime

Zebrafish are fed with a combination of live and processed feeds. Live feeds comprise *Paramecium caudatum* and decapsulated *Artemia salina. A. salina* dehydrated cysts (Brine Shrimp Cysts, Basic 230, Grade 6 from Zebrafish Management Ltd.) are decapsulated by bleach, according to the Zebrafish International Resource Center (ZIRC) protocol, available in https://zebrafish.org/documents/protocols.php. *P. caudatum* culture has been maintained in-house since 2009 following the protocol described in Nusslein-Volhard and Dahm.[1] Processed diets consist of Mucedola 0.2–0.3 (Mucedola®) and Gemma Micro 300 (Skreting®). Larvae between 5–8 days post-fertilization (dpf) are fed once a day with *P. caudatum* and twice a day with Mucedola 0.2–0.3. Larvae from 8 to 20 dpf are fed thrice a day; twice with live feeds (progressively weaned of *P. caudatum*, while gradually increasing decapsulated *A. salina)* and supplemented once a day with dry feed Mucedola 0.2–0.3. From 20 to 90 dpf, animals are fed twice a day with *A. salina* and once a day with dry food (Mucedola 0.2–0.3 until 30 dpf and Gemma micro 300 onward). Adult fish are fed once a day with *A. salina* and once with Gemma micro 300 (active breeders are fed thrice/day). Fish are fed to satiation.[2] Mucedola composition is as follows: 56% crude protein, 13.5% crude oil and fats, 0.5% crude fibers, 10.5% crude ash, 1.8% phosphorus, 2.9% calcium, and 0.9% sodium. Gema Micro composition is as follows: 55% protein, 15% lipids, 13.5% ash, 5% fiber, 2% phosphorus, and 14.3% total n-3 HUFA of fat.

Animal Density and Nursery

Zebrafish lifestages are defined as larval from 2 to 30 dpf; juvenile from 30 dpf until 90 dpf, a time point when most animals have reached sexual maturation; and adults from 90 dpf onward. Larvae are reared at a density of 10–12 fish/L and juvenile and adult fish at 5–10 fish/L.[3,4] The main recirculating system has a dedicated area for nursery where larvae are housed until they become adults. At 5 dpf, larvae are transferred from Petri dishes to 3.5 L tanks filed with 500 mL homemade E3 medium, fed with 1–2 mL/larvae of *P. caudatum*, and placed in static conditions for 24 h.[1] On the following day, the flow is turned on (1 drop/second) and increased gradually until animals reach juvenile stage. During this period, tanks are gently siphoned once a week with a hose or plastic Pasteur pipette to remove debris.

DNA Extraction from Suspect *Mycobacterium* spp. Infection Cases

The extraction of total DNA from paraffin-embedded tissues is performed with the High Pure PCR Template Preparation Kit (Roche), following the manufacturer's instructions for this type of samples. A step of mechanical disruption, before applying Proteinase K, is included using a bead-beating protocol (with zirconium beads), consisting of 6.5 ms^{-1} cycles for 45 s, repeated twice, in the FastPrep FP120 Bio101 apparatus (Savant Instruments). Genomic DNA suspensions are stored at –20°C until further use. DNA concentration and purity are estimated by measuring the absorbance at 260 nm (A260) and determining the A260/A280 and A260/A230 ratios, respectively, using a NanoDrop 1000 spectrophotometer (NanoDrop).

Identification of Mycobacteria to the Species Level by Polymerase Chain Reaction and Restriction Enzyme Analysis

Detection of mycobacteria nucleic acids is performed by polymerase chain reaction (PCR) targeting a 439-bp fragment of the genus-specific gene encoding the 65 kDa heat shock protein (Hsp65). Identification to the species level is completed by restriction fragment length polymorphism analysis using *Bst*EII and *Hae*III restriction enzymes,[5] which allows identification of several tuberculous and nontuberculous (NTM) mycobacterial species. Genomic DNA extracted from purified reference and clinical bacterial strains of *Mycobacterium tuberculosis* complex, identified to the species level,[6] and NTM, are included as positive controls in each PCR assay. A reaction with nuclease-free water instead of DNA template is included as negative control. Another genus-specific PCR with identification to the species level of slow growing and rapidly growing mycobacteria is employed complementarily using the 16S–23S ribosomal RNA gene (rDNA) spacer (Internal Transcribed Spacer, ITS) as target.[7] The speciation algorithm involves the estimation of variable species-specific PCR product sizes (205–318 bp amplicons), together with *Hae*III and *Cfo*I (first step), and additional *Taq*I, *Msp*I, *Dde*I, or *Ava*II (second step) restriction analyses.

Mycobacterium spp. 16S rDNA Amplification by Real-Time PCR

Real-time PCR reactions aiming the amplification of mycobacterial 16S rDNA (149-bp fragment),[8] in a total volume of 20 μL containing 1× SsoAdvanced Universal Probes Supermix (Bio-Rad), 0.4 μM of each primer F_16SrDNA (5′-CCG CAA GGC TAA AAC TCA AA-3′) and R_16SrDNA (5′-TGC ACA CAG GCC ACA AGG GA-3′), 0.15 μM of TaqMan probe P_16SrDNA (TET-TCG ATG CAA CGC GAA GAA CCT TAC-BHQ1), nuclease-free water (GIBCO), and 5 μL of the extracted DNA template. The probe is labeled with tetrachlorofluorescein (TET) fluorophore and BHQ1 quencher. Positive and template-free negative controls are included in each reaction.

The thermal cycling conditions are as follows: 1 cycle at 95°C for 2 min, followed by 45 cycles at 95°C for 5 s and 60°C for 10 s. All samples are tested thrice and considered positive if at least one of the amplification reactions yields positive. Thermal cycling, fluorescent data collection, and data analysis are carried out in a CFX96 (Bio-Rad) detection system real-time PCR instrument.

Supplementary Table S1. Health Program Checklist

	Test method
Signs	
Abnormal swimming	Visual
Anal prolapsed	Visual
Clamped fins	Visual
Cutaneous mass	Visual
Distended coelomic cavity	Visual
Distended coelomic cavity + scale protrusion	Visual
Dorsal scale protrusion	Visual
Dyspnea	Visual
Emaciation	Visual
Group mortality	Visual
Negative buoyancy	Visual
Positive buoyancy	Visual
Skin congestion	Visual
Skin hemorrhage	Visual
Spinal curvature	Visual
Subcutaneous emphysema	Visual
Lesions	
Aerocystitis	Necropsy/Histology
AFB negative granuloma	Histology
Branchitis	Histology
Cardiac dilatation	Necropsy/Histology
Cutaneous ulcer	Visual/Necropsy/Histology
Egg binding	Necropsy/Histology
Exophthalmitis	Visual/Necropsy/Histology
Gill air emboly	Necropsy/Histology
Gill hyperplasia	Histology
Gill telangiectasia/aneurism	Necropsy/Histology
Intestinal dilatation	Necropsy/Histology
Macroscopic granuloma	Necropsy
Muscle fiber atrophy	Histology
Neoplasia	Histology
Opaque swim bladder	Necropsy
Ovarian inflammation	Histology
Coelomitis	Necropsy/Histology
Overinflated swim bladder	Necropsy/Histology
Splenomegaly	Necropsy/Histology
Supersaturation	Necropsy/Histology
Testicular hypertrophy	Necropsy/Histology
Thyroid hypertrophy	Visual/Necropsy/Histology
Pathogens	
Bacteria	
Acid-fast bacteria	Histology
Flavobacter-like-bacteria	Necropsy/Histology
Gram negative bacteria	Histology
Parasites	
Capilarid nematodes	Necropsy/Histology
Cestodes	Necropsy/Histology
Digenetic trematodes	Necropsy/Histology
Ichthyophthirius multifiliis	Necropsy/Histology
Monogenetic trematodes	Necropsy/Histology
Nematode eggs	Necropsy/Histology
Piscinoodinium spp.	Necropsy/Histology
Pseudoloma neurophilia	Histology
Tetrahymena spp.	Necropsy/Histology
Mycology	
Fungal hyphae	Necropsy/Histology
Macroscopic hyphae	Visual

Full list of clinical signs, histopathological lesions and pathogens detected by listed methods.
AFB, acid-fast bacteria.

Supplementary Table S2. Quarantine Program Results

Clinical finding		Results	
		#pos/#test	freq (%)
Signs	Emaciation	10/181	5.5
	Abnormal swimming	6/181	3.3
	Skin hemorrhage	4/181	2.2
	Spinal curvature	4/181	2.2
	Dorsal scale protrusion	3/181	1.7
	Distended coelomic cavity	2/181	1.1
	Group mortality	2/181	1.1
	Dyspnea	1/181	0.6
	Skin congestion	1/181	0.6
	Positive buoyancy	1/181	0.6
Lesions	Aerocystitis	8/181	4.4
	Coelomitis	8/181	4.4
	Cutaneous ulcer	8/181	4.4
	Neoplasia	5/131	3.8
	AFB negative granuloma	3/131	2.3
	Intestinal dilatation	3/181	1.7
	Branchitis	2/131	1.5
	Muscle fiber atrophy	2/131	1.5
	Ovarian inflammation	2/131	1.5
	Gill hyperplasia	1/131	0.8
	Egg binding	1/181	0.6
	Gill air emboly	1/181	0.6
Pathogens	Pseudoloma neurophilia	23/131	17.6
	Acid-fast bacteria	8/131	6.1
	Fungal hyphae	2/181	1.1
	Macroscopic hyphae	1/181	0.6
	Capilarid nematodes	1/181	0.6
	Nematode eggs	1/181	0.6

pos, positive; test, tested; freq, frequency.

Supplementary References

1. Nusslein-Volhard C, Dahm R: Zebrafish: A Practical Approach. Oxford University Press, Oxford, England, 2002.
2. Lawrence C. The husbandry of zebrafish (*Danio rerio*): A review. Aquaculture 2007;269:1–20.
3. Harper C, Lawrence C: The Laboratory Zebrafish. CRC Press, Boca Raton, FL, 2011.
4. Castranova D, Lawton A, Lawrence C, Baumann DP, Best J, Coscolla J, *et al.* The Effect of Stocking Densities on Reproductive Performance in Laboratory Zebrafish (*Danio rerio*). Zebrafish 2011;8:141–146.
5. Telenti A, Marchesi F, Balz M, Bally F, Bottger EC, Bodmer T. Rapid identification of mycobacteria to the species level by polymerase chain reaction and restriction enzyme analysis. J Clin Microbiol 1993;31:175–178.
6. Cunha MV, Monteiro M, Carvalho P, Mendonça P, Albuquerque T, Botelho A. Multihost tuberculosis: insights from the portuguese control program. Vet Med Int 2011;2011:795165.
7. Roth A, Reischl U, Streubel A, Naumann L, Kroppenstedt RM, Habicht M, *et al.* Novel diagnostic algorithm for identification of mycobacteria using genus-specific amplification of the 16S-23S rRNA gene spacer and restriction endonucleases. J Clin Microbiol 2000;38:1094–1104.
8. Costa P, Amaro A, Ferreira AS, Machado D, Albuquerque T, Couto I, *et al.* Rapid identification of veterinary-relevant Mycobacterium tuberculosis complex species using 16S rDNA, IS6110 and Regions of Difference-targeted dual-labelled hydrolysis probes. J Microbiol Methods 2014;107:13–22.
